# The Potato Aphid Salivary Effector Me47 Is a Glutathione-*S*-Transferase Involved in Modifying Plant Responses to Aphid Infestation

**DOI:** 10.3389/fpls.2016.01142

**Published:** 2016-08-03

**Authors:** Graeme J. Kettles, Isgouhi Kaloshian

**Affiliations:** Department of Nematology, University of California, RiversideRiverside, CA, USA

**Keywords:** effector, potato aphid, glutathione-*S*-transferase, GST, secretome

## Abstract

Polyphagous aphid pests cause considerable economic damage to crop plants, primarily through the depletion of photoassimilates and transfer of viruses. The potato aphid (*Macrosiphum euphorbiae*) is a notable pest of solanaceous crops, however, the molecular mechanisms that underpin the ability to colonize these hosts are unknown. It has recently been demonstrated that like other aphid species, *M. euphorbiae* injects a battery of salivary proteins into host plants during feeding. It is speculated that these proteins function in a manner analagous to secreted effectors from phytopathogenic bacteria, fungi and oomycetes. Here, we describe a novel aphid effector (Me47) which was identified from the potato aphid salivary secretome as a putative glutathione-*S*-transferase (GST). Expression of Me47 in *Nicotiana benthamiana* enhanced reproductive performance of green peach aphid (*Myzus persicae*). Similarly, delivery of Me47 into leaves of tomato (*Solanum lycopersicum)* by *Pseudomonas* spp. enhanced potato aphid fecundity. In contrast, delivery of Me47 into *Arabidopsis thaliana* reduced GPA reproductive performance, indicating that Me47 impacts the outcome of plant–aphid interactions differently depending on the host species. Delivery of Me47 by the non-pathogenic *Pseudomonas fluorescens* revealed that Me47 protein or activity triggers defense gene transcriptional upregulation in tomato but not *Arabidopsis*. Recombinant Me47 was purified and demonstrated to have GST activity against two specific isothiocyanates (ITCs), compounds implicated in herbivore defense. Whilst GSTs have previously been associated with development of aphid resistance to synthetic insecticides, the findings described here highlight a novel function as both an elicitor and suppressor of plant defense when delivered into host tissues.

## Introduction

Aphids are a large family of hemipteran insects that feed from the vasculature tissue of plants. They feed by inserting their flexible hypodermal needle-like mouthpart or stylets into plant tissue and navigate mostly between cells until they puncture the phloem tissue and feed from the sugar-rich sap (Tjallingii and Esch, 1993; Tjallingii, 2006). Both during initial probing and feeding, aphids secrete watery saliva from their stylets (Tjallingii, 2006). It is known that salivary secretions from aphids play important roles in the establishment and maintenance of successful feeding sites (Will et al., 2007). For example, phloem-plugging in fava bean is dependent on the expansion of forisomes in sieve elements. This process can be inhibited by application of aphid salivary extracts (Will et al., 2007). Saliva from numerous aphid species is known to contain a complex mix of proteins (Harmel et al., 2008; Carolan et al., 2009, 2011; Cooper et al., 2011; Nicholson et al., 2012; Rao et al., 2013; Nicholson and Puterka, 2014; Vandermoten et al., 2014; Chaudhary et al., 2015). It is speculated that salivary proteins act in ways similar to protein effectors from plant microbial pathogens. That is, to inhibit or suppress the activation of host immune processes and enable successful colonization. Whilst the salivary protein complement of several aphid species is now known, functional characterization of individual proteins has extended to just a handful of examples (Jaouannet et al., 2014; Kaloshian and Walling, 2016). The pea aphid *Acyrthosiphon pisum* protein C002 is injected into fava bean during feeding and is required for effective feeding behavior (Mutti et al., 2008). Two proteins (Mp10, Mp42) were identified from the green peach aphid *Myzus persicae* that reduced aphid performance when transiently expressed in *Nicotiana benthamiana* by *Agrobacterium tumefaciens* (Bos et al., 2010). Mp10 induces chlorosis/cell death in *N. benthamiana* suggesting direct recognition of this salivary protein through mechanisms that are distinct from Mp42 (Bos et al., 2010; Rodriguez et al., 2014). Further effectors from *M. persicae* (Mp1, Mp2, Mp55, Mp56, Mp57, and Mp58) have been reported that have various impact on aphid fecundity when either transiently or stably expressed in hosts (Pitino and Hogenhout, 2013; Elzinga et al., 2014). However, the molecular functions of these proteins are unknown. Two effectors (Me10, Me23) have to date been identified from the potato aphid *Macrosiphum euphorbiae* (Atamian et al., 2013). Me10 and Me23 both increase aphid performance when delivered by the bacterium *Pseudomonas syringae* type three secretion system (T3SS) into *N. benthamiana*; however, only Me10 had a similar effect when introduced into tomato (*Solanum lycopersicum*) leaves using the same delivery method (Atamian et al., 2013). As for other aphid effectors, the specific performance-enhancing activities of Me10 and Me23 are unknown.

The mechanisms by which plants defend themselves against aphid attack are wide-ranging. Preformed physical defenses include barriers such as trichomes, waxy cuticles and oily secretions to discourage aphid settling. There are also inducible changes that occur following the onset of aphid feeding. These include transcriptional modifications, generation of reactive oxygen species (ROS), callose deposition and the production of toxic phytoalexins (Moran et al., 2002; Martinez de Ilarduya et al., 2003; De Vos et al., 2005; Kusnierczyk et al., 2008; Louis et al., 2010; Kettles et al., 2013). The perception of microbial plant attackers, lately shown for aphids as well, has been conceptualized in a multi-layered model of plant defense (Jones and Dangl, 2006; Kaloshian and Walling, 2016). In the first instance, immune recognition of conserved Pathogen-Associated Molecular Patterns (PAMPs) results in PAMP-triggered immunity (PTI) which in most cases is enough to prevent infection or colonization. Only if the pathogen or pest has means to overcome PTI and suppress these inducible changes, typically through the action of proteinaceous effectors or other metabolites, can disease or colonization be achieved.

In order to overcome powerful host defenses, aphids must evolve ways of either suppressing the activation of plant immune processes or detoxifying the resulting chemical assault mounted by the host. Glutathione-*S*-transferases (GSTs) are a class of detoxification enzyme found throughout the eukaryotic kingdom that catalyzes conjugation of reduced glutathione (GSH) to both natural and synthetic xenobiotics (Li et al., 2007). Specifically for insect pests of plants, they are often grouped with classes of other detoxifying enzymes such as cytochrome P450s and carboxy/cholinesterases (Li et al., 2007; Ramsey et al., 2010) and have been linked to the development of resistance against chemical insecticides (Vontas et al., 2001, 2002). In addition to their role in insecticide resistance, GSTs are assumed to protect insects from xenobiotics encountered in nature. Aphid GSTs are induced when feeding on resistant plants (Bansal et al., 2014) or when fed on toxins in artificial diet (Francis et al., 2005). It has been speculated that diversity of GSTs may contribute to host-range of aphids due to capacity to metabolize a greater variety of host toxins (Ramsey et al., 2010). Study of GSTs in insect pests has largely focussed on those present in gut tissue and their interaction with compounds ingested during feeding. The role of GSTs deployed out on or into plant tissues and their interaction with host immune systems is unexplored.

Recent bioinformatic and proteomic analyses of the *M. euphorbiae* salivary secretome (Atamian et al., 2013; Chaudhary et al., 2014, 2015) revealed the presence of a single putative GST in aphid saliva. In this investigation, we describe the functional characterization of this candidate effector which we have named Me47. The impact of Me47 expression on performance of two aphid species across three different hosts was examined. Additionally, we find an inverse correlation between Me47-dependent activation of defense responses and aphid performance. Finally, we present evidence of substrate specificity of Me47 which helps explain the role of this GST in plant–aphid interactions.

## Materials and Methods

### Phylogenetic Analysis and Secretion Signal Prediction

Glutathione sequences from *A. pisum* (Ramsey et al., 2010) were obtained from AphidBase 2.1 (INRA). *M. persicae* GST sequences were recovered from Myzus DB (INRA) by low stringency (*E* < 0.1) Blastp analysis of both *M. persciae* clone O and clone G006 genomes using Me47 sequence as query. Me47 coding sequence was aligned to GST sequences from *A. pisum* and *M. persicae* (both clones G006 and O; Supplementary Table S2) using ClustalW and displayed using a Neighbor-Joining tree with 100 bootstrap replicates using Geneious software (Biomatters). *M. persicae* protein identifiers are presented as MpG006 or MpO to indicate clonal origin.

The predicted amino acid sequences of the GSTs were subjected to *de novo* signal peptide prediction analysis using SignalP 4.1 and TargetP 1.1 programs (Emanuelsson et al., 2000; Petersen et al., 2011). For SignalP a Hidden Markov model scores higher than 0.45 was used. For TargetP predictions were determined by predefined set of cutoffs that yielded specificity >0.95 on the test sets.

### Me47 Cloning and Bacterial Transformation

Me47 coding sequence lacking the secretion signal was amplified from 100 ng of potato aphid cDNA using primers attB1 Me47-F and attB2 Me47-R (Supplementary Table S1) and high-fidelity Phusion polymerase (New England Biolabs) with the following thermocycle (30 s at 95°C, 30 s at 55°C, 30 s at 72°C × 30 cycles). The attB-flanked Me47 PCR product was recombined into pDONRzeo using BP clonase (Invitrogen) following the manufacturer’s instructions. Me47 was sequence verified by Sanger sequencing before subsequent shuttling into the destination vectors pEARLEYGATE100 for *in planta* Agroexpression (Earley et al., 2006), pVSP_*Ps*SPdes for bacterial delivery in tomato and *Arabidopsis* (*Arabidopsis thaliana*; Rentel et al., 2008) and pDEST17 (Invitrogen) for recombinant protein expression using LR clonase (Invitrogen). For initial cloning, electrocompetent DH5α cells were used for all transformations and *Agrobacterium* strain GV3101 was used for Agroexpression following standard procedures

### Plant Materials and Aphid Colonies

Tomato cultivars (cv.) UC82B and Moneymaker, *N. benthamiana*, tobacco (*Nicotiana tabacum)* NC-95, mustard India, and *Arabidopsis* Col-0 were used. Seeds were planted directly into autoclaved soil or transplanted after seeding into soil. Plants were maintained in growth rooms at 22–24°C with 16 h day length and 200 μmol m^-2^ s^-1^ light intensity. Solanaceous plants were weekly fertilized with MiracleGro (18-18-21; Stern’s MiracleGro Products).

Colonies of the parthenogenetic *M. euphorbiae* were reared on tomato cv. UC82B, while *M. persicae* was reared on tobacco NC-95 or mustard plants. The colonies were maintained in insect cages in a pesticide-free greenhouse at 22–26°C supplemented with light for 16 h day length. One-day old age synchronized *M. euphorbiae* adults were produced as described by Bhattarai et al. (2007).

### Aphid Performance Assays

To assess *M. persicae* performance on *N. benthamiana, Agrobacterium* carrying either pEARLEYGATE100-GFP or pEARLEYGATE100-Me47 were grown in LB supplemented with appropriate antibiotics for 36 h at 28°C. Cells were washed thrice and resuspended in infiltration buffer (10 mM MgCl_2_, 10 mM MES, 100 μM acetosyringone, pH 5.6) to an OD_600_ = 0.3. Bacteria were infiltrated into fully expanded leaves using a needleless syringe. After 24 h, four adult *M. persicae* were applied to infiltrated leaves within clip cages and left to produce a population of age-synchronized nymphs (day 0). After 48 h, all adults and excess nymphs were removed leaving five nymphs on each leaf (day 2). Nymphs were allowed to feed for two further days before being transferred to a second set of plants which had been similarly infiltrated 24 h previously (day 4). Aphids were allowed to feed from the second set of leaves for 4 days, before transfer to a final set of plants infiltrated 24 h previously (day 8). Experiments were terminated on day 12. This method allowed nymphs to mature to adulthood whilst being continuously exposed to high levels of transgene expression. Aphids typically began production of the next generation of nymphs on day 8. Aphid counts were made daily on days 8–12 and nymphs were continuously removed, such that each count represented fecundity over a 24 h period. Counts from all days were pooled for analysis. The experiment was conducted three times with similar results. Comparison of aphid fecundity on GFP-expressing and Me47-expressing leaves was assessed by two-tailed *t*-test.

To assess *M. euphorbiae* performance on tomato, GUS or Me47 was delivered by either semi-virulent *P. syrinagae* pv. *tomato* (*Pst*) DC3000 ΔAvrPto ΔAvrPtoB or non-pathogenic *P. fluorescens* (*Pfo*) EtHAn engineered with a T3SS (Thomas et al., 2009). In both systems, bacteria were cultured on Kings B plates with appropriate antibiotics for 36 h at 30°C. Cells were washed from plates in 10 mM MgCl_2_ and resuspended to a density of 1 × 10^3^ CFU/mL in infiltration buffer (1 mM MgCl_2_, 0.02% Silwet L-77) in 2.5 L total volume. Whole plants were upturned and submerged in infiltration buffer, placed in a vacuum chamber and infiltrated for 2 min at 20 inHg. Plants were immediately transferred to a growth cabinet and allowed to recover overnight. At 24 h post infection (hpi), 10 mature age-synchronized adult *M. euphorbiae* were applied to the leaves of each plant with a fine paintbrush. Counts of both the surviving adults and newly born nymphs were made daily for 5 days and all nymphs were removed each day such that each count represented fecundity over a 24 h period. The counts from all days were pooled for analysis and each experiment was conducted three times with similar results. Comparison of aphid fecundity on GUS-expressing and Me47-expressing leaves was assessed by two-tailed *t*-test.

For *Arabidopsis* performance assays, *Pfo* EtHAn strain was prepared as for the tomato assay except that leaves were individually syringe-infiltrated rather than whole-plant submersion infiltration. At 24 hpi, single age-synchronized adult *M. euphorbiae* were applied to the center of each rosette and the whole plant caged. Counts of newly born nymphs were made daily for 5 days as described for the tomato assay. The experiment was conducted three times (**Figure 2B**; Supplementary Figure S3B) and results were analyzed as for the tomato assay.

### Induction of Plant PAMP Responses in Tomato and *Arabidopsis*

High-dose *Pfo* inoculum was prepared following the method described above, with the exception that bacteria were infiltrated at OD = 0.01 (∼1 × 10^6^ CFU/mL; Nguyen et al., 2010) compared to the lower dose used for aphid performance assays. Following infiltration, plants were returned to growth conditions until sample harvest at 6 hpi. Experiments were conducted twice with three biological replicates per experiment. Expression data from both experiments were combined and analyzed together. Comparisons of expression levels of defense-related genes in GUS-expressing and Me47-expressing leaves were made using a two-tailed *t*-test.

### qRT-PCR

Leaf tissues from *Pfo*-infiltrated tomato or *Arabidopsis* plants were harvested at 6 hpi and snap frozen. Samples were ground in collection tubes using pellet pestles (Sigma) and total RNA extracted using Trizol (Invitrogen) as per the manufacturer’s instructions. The 260/280 ratios of all samples were checked using a Nanodrop spectrophotometer and all were between 1.8 and 2.1 μg of total RNA was treated with DNaseI (NEB) and samples were subsequently tested for gDNA contamination by PCR amplification using either *UBI3* (tomato) or *PEX4* (*Arabidopsis*) primer pairs. First strand cDNA synthesis was performed using the SuperScript III kit (Invitrogen). cDNA was diluted 1:10 with dH_2_O prior to qRT-PCR and 1 μl of this dilution was used per reaction.

Duplicate reactions for each sample/primer-pair combination were conducted using clear 96-well PCR plates (Bio-Rad) and iQ SYBR Green Supermix (Bio-Rad). Reactions were carried out using an iCycler real-time PCR system (Bio-Rad) using the following thermocycle (5 min at 95°C followed by 30 s at 95°C, 30 s at 58°C, 30 s at 72°C × 40 cycles). Relative expression values for defense-related genes were calculated using the formula 2^-Δ^*^C^*^t^ (Pfaﬄ, 2001) relative to the *TIP41* reference gene (tomato) or *PEX4* (*Arabidopsis*). Expression values were rescaled for presentation such that the buffer treatment is equal to 1.

### Protein Purification and Western Blot Analysis

*Escherichia coli* ArcticExpress cells (Agilent) carrying the pDEST17-Me47 construct were grown in LB media at 37°C to an OD_600_ of 0.8. Recombinant Me47 production was induced by addition of 1 mM IPTG followed by incubation at 12°C for 16 h. Cells were recovered by centrifugation, resuspended in chilled lysis buffer (300 mM NaCl, 50 mM sodium phosphate, pH7.2) and lysed using sonication (6 × 30 s pulses). The soluble protein fraction was collected and incubated with Ni-NTA agarose beads (Qiagen) for 1 h at 4°C with gentle agitation. Non-specifically bound proteins were removed with four washes of lysis buffer containing 25 mM imidazole. His-tagged Me47 protein was eluted with two washes of lysis buffer containing 250 mM imidazole. Aliquots were taken at all stages of the purification process and protein content assessed by Bradford assay. Twenty micrograms of all samples were separated by SDS-PAGE using a 12% acrylamide gel. To confirm the identity of purified His-tagged Me47, protein was transferred to nitrocellulose membrane and probed with HisProbe-HRP conjugate antibody (Santa Cruz Biotechnology) in PBST with 2% milk powder. Signal was detected by Amersham ECL Prime Western Blotting Detection Reagent (GE Healthcare) and imaged using X-ray film.

### Glutathione Depletion Assay

Fractions of N-terminal His-tagged Me47 protein were pooled and imidazole removed by buffer exchange using PBS (pH 6.5) and PD10 buffer exchange columns (GE Healthcare). N-terminal His-tagged GroEL was prepared using the same method. To assess activity against ITC substrates, 2 μg of each protein treatment (equine liver GST, His-Me47, His-GroEL) were incubated in the presence of 50 μM glutathione and 200 μM of three ITCs (AITC, BITC, and PEITC) at pH 7.0 for 20 min at room temperature. Buffer-only control reactions with no protein treatment were also included. The concentration of free glutathione remaining in each reaction was assessed using the Glutathione Assay Kit (Sigma) following the manufacturer’s instructions.

### ROS Burst Assay

GFP, Me47, and Mp10 (Bos et al., 2010) were expressed in *N. benthamiana* leaf tissue following Agroinfiltration with GV3101 containing pEARLEYGATE100-GFP, pEARLEYGATE100-Me47, or pEARLEYGATE100-Mp10 as described above. At 2 dpi, 2 mm × 2 mm leaf squares from the Agroinfiltrated leaves were cut using a razor blade and soaked overnight in dH_2_O. Leaf squares were subsequently exposed to flg22 (100 nM) in a luminol-based assay (Chaudhary et al., 2014) and luminosity was recorded using a Mithras LB 940 Multimode Reader luminometer (Berthold Technologies) for 25 min. For assays to test elicitor activity of Me47, naive *N. benthamiana* leaf disks were prepared as described above before exposure to reaction cocktail containing flg22 (100 nM), Me47 (1.5 μM) or PBS as negative control.

## Results

### Identification and Phylogenetic Analysis of Me47

**FIGURE 1:**
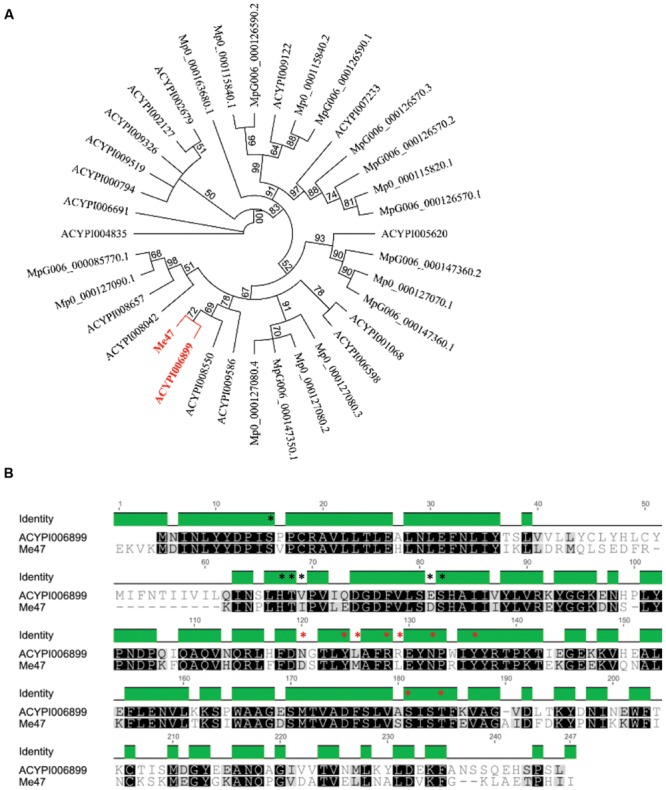
**Phylogenetic analysis of Me47 relative to *A. pisum* and *M. persicae* GST sequences. (A)** Predicted Me47 amino acid sequences were aligned with *M. persicae* (both clones G006 and O) and *A. pisum* GSTs using ClustalW and presented as a phylogenetic tree using the Neighbor-joining method (Geneious, 100 bootstrap replicates). Me47 and the closest *A. pisum* ortholog (ACYPI006899) are highlighted in red. **(B)** Alignment of Me47 with ACYPI006899. Residues forming the GSH binding site (G site; black) and substrate binding pocket (H site; red) are marked with asterisks.

The identification of the proteinaceous components of *M. euphorbiae* saliva and the correlation with salivary gland EST data has been described previously (Atamian et al., 2013; Chaudhary et al., 2014, 2015). This analysis revealed the presence of a single protein (contig Me_WB05003; Me47), encoding 261 amino acids, with predicted GST activity based on homology to known enzymes of this type. To characterize Me47 and to perform phylogenetic analysis, using BLASTp at low stringency (*E* < 0.1), we identified the GST homologs from the two aphid species with publically available genome sequences. These are the legume specialist *A. pisum* and the generalist *M. persicae* with genome sequences for two distinct clones (The International Aphid Genomics Consortium [TIAGC], 2010; Myzus DB). These analyses identified 17 GSTs (AphidBase 2.1; Ramsey et al., 2010) from *A. psium* and nine GSTs from each of the *M. persicae* clones O and G006 (Myzus DB). We identified several alternate spliced forms of some of these GSTs and only one representative of these was included in further analysis. Phylogenetic analysis of Me47 coding sequence relative to the GST predicted proteins identified from *A. pisum* and *M. persicae* revealed that Me47 is more similar to GSTs from *A. pisum* (**Figure 1A**). *A. pisum* has three different classes of GST and the closest homolog to Me47 is the delta-class GST ACYPI006899 which encodes a 241 amino acid protein (Chelvanayagam et al., 2001; Ramsey et al., 2010). Direct comparison between Me47 and ACYPI006899 showed 62% identity at the amino acid level (**Figure 1B**) with conservative or semi-conservative substitutions at 2/6 positions of the GSH-binding site (G site; **Figure 1B** black asterisks) and 2/9 positions of the substrate-binding pocket (H site; **Figure 1B** red asterisks).

Since Me47 peptides were detected in the *M. euphorbiae* saliva (Chaudhary et al., 2014, 2015), we investigated the presence of a secretion signal peptide cleavage site in the predicted Me47 protein. Using SignalP, the presence of a 28 amino acid secretion signal was identified in Me47 confirming secretion of this GST in aphid saliva (Petersen et al., 2011). Curiously, ACYPI006899 does not contain a secretion signal cleavage site predicted by SignalP. Indeed, of the 36 aphid GSTs in this analysis, only three (Me47, ACYPI009586 and MpO_000127080.4) contain putative canonical secretion signal cleavage sites. Secretion of proteins could also be predicted by TargetP in the absence of a secretion signal peptide cleavage site (Emanuelsson et al., 2000). Using TargetP with the remaining *M. persicae* and *A. pisum* GSTs, predicted secretion for two additional *A. pisum* GSTs (ACYPI006899 and ACYPI006691) including the Me47 homolog (ACYPI006899) was identified. Taken together this information indicates that aphid GSTs have evolved different mechanisms for secretion and that their secretion into either extracellular spaces or saliva is relatively uncommon.

### Me47 Modifies Aphid Performance in Multiple Fecundity Systems

**FIGURE 2:**
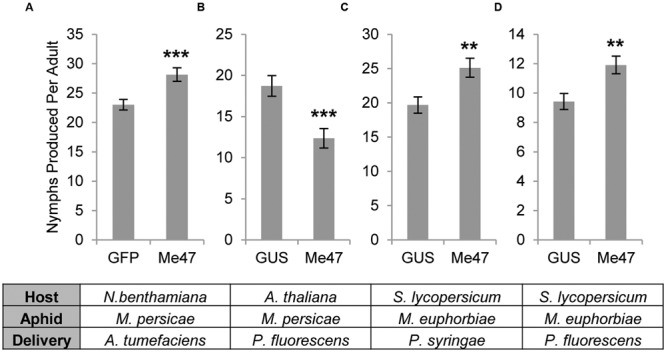
**Me47 changes aphid reproductive performance. (A)** *M. persicae* fecundity assessed on *N. benthamiana* transiently expressing either GFP or Me47 by Agroinfiltration. **(B)** *M. persicae* fecundity assessed on *A. thaliana* infected with either *Pfo* EtHAn (pVSP-GUS) or *Pfo* EtHAn (pVSP-Me47). **(C)** *M. euphorbiae* fecundity assessed on tomato cv. Moneymaker infected with either *Pst* DC3000 Δ*AvrPto*/Δ*AvrPtoB* (pVSP-GUS) or *Pst* DC3000 Δ*AvrPto*/Δ*AvrPtoB* (pVSP-Me47). **(D)** *M. euphorbiae* fecundity assessed on tomato cv. Moneymaker infected with either *Pfo* EtHAn (pVSP-GUS) or *Pfo* EtHAn (pVSP-Me47). ^∗∗^*P* < 0.01 and ^∗∗∗^*P* < 0.001 as determined by two-tailed *t*-test. Data from single experiments presented and data from additional experiments are shown in Supplementary Figure S3.

To examine the role of Me47 during aphid colonization, we used Agrobacterium-mediated transient expression to express Me47 in leaf tissue of *N. benthamiana*. As *M. euphorbiae* does not reproduce successfully on *N. benthamiana*, we assessed the fecundity of *M. persicae*, which is able to feed on this host, over a 12-day period in an assay similar to experiments conducted previously (Bos et al., 2010; Atamian et al., 2013). In these experiments, we found that *M. persicae* fecundity was significantly increased on Me47-expressing leaves compared to GFP-expressing control leaves (**Figure 2A**, *P* < 0.001, Supplementary Figure S3A). This indicates that Me47 may function as a suppressor of plant immunity to enhance aphid colonization of tobacco.

To assess the role of Me47 in wider plant–aphid interactions, we transformed the semi-virulent bacterial strain *Pst* DC3000 Δ*AvrPto*/Δ*AvrPtoB* with the construct pVSP_*Ps*SPdes Me47 (pVSP-Me47). Whole tomato plants were vacuum-infiltrated with this semi-virulent inoculum. Using this method, the aphid protein of interest is delivered into tomato leaf cells via the bacterial T3SS, allowing performance of *M. euphorbiae* to be assessed on its natural host (Atamian et al., 2013). In these trials, *M. euphorbiae* fecundity was significantly increased on plants infected with *Pst* DC3000 Δ*AvrPto*/Δ*AvrPtoB* (pVSP-Me47) relative to those infected with the *Pst* DC3000 Δ*AvrPto*/Δ*AvrPtoB* (pVSP-GUS) control (**Figure 2C**, *p* < 0.01, Supplementary Figure S3C). This indicates that Me47 can function as a pathogenicity determinant in at least two host plant species with impact on two distinct aphid pests. However, as *Pst* DC3000 Δ*AvrPto*/Δ*AvrPtoB* possesses its own effector complement and is semi-virulent to tomato, we chose to assess the effect of Me47 delivery in the absence of other pathogen effectors. For this experiment, we used the non-pathogenic *Pfo* EtHAn strain (Thomas et al., 2009), which has been engineered to express the T3SS. This strain was transformed with the same constructs used in experiments described for *Pst* DC3000 Δ*AvrPto*/Δ*AvrPtoB*. In fecundity assays, *M. euphorbiae* performed significantly better on tomato infected with *Pfo* EtHAn (pVSP-Me47) compared to a *Pfo* EtHAn (pVSP-GUS) control (**Figure 2D**, *p* < 0.01, Supplementary Figure S3D). This confirmed our previous result in tomato, and indicates that the choice of *Pseudomonas* species for delivery of Me47 has minimal impact on the role of this protein in modifying *M. euphorbiae* fecundity on tomato. Finally, we used *Pfo* EtHAn with the same constructs to assess *M. persicae* fecundity on *Arabidopsis*. Interestingly in these experiments, *M. persicae* fecundity was significantly reduced on plants infected with *Pfo* EtHAn (pVSP-Me47) relative to the *Pfo* EtHAn (pVSP-GUS) control (**Figure 2B**, *p* < 0.001, Supplementary Figure S3B). This indicates that in specific host–aphid interactions, Me47 can have a host-dependent deleterious impact on aphid fecundity.

### Me47 Induces PAMP-Responsive Genes in Tomato But Not in *Arabidopsis*

**FIGURE 3:**
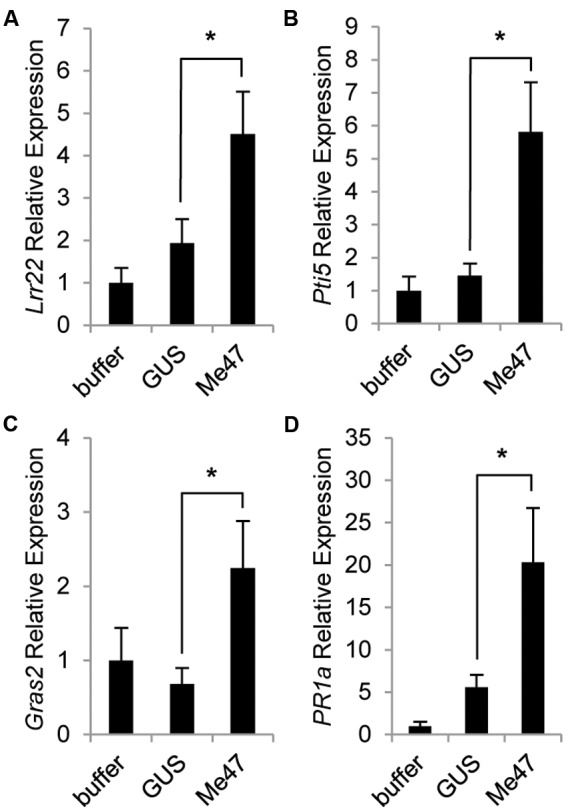
**Me47 induces defense genes in tomato.** Tomato cv. Moneymaker was infiltrated with buffer-only, *Pfo* EtHAn (pVSP-GUS) or *Pfo* EtHAn (pVSP-Me47) and leaves harvested at 6 hpi. Expression analysis of genes involved in PTI (*Lrr22, Pti5, Gras2*) **(A–C)** and salicylic acid (SA)-dependent defense (*PR1a*) **(D)** conducted by qRT-PCR. ^∗^*P* < 0.05 as determined by two-tailed *t*-test. Bars represent means and standard error across six biological replicates from two independent experiments. Buffer treatment rescaled to 1 for presentation.

Our data indicated that in some experimental systems, Me47 made a positive contribution to aphid fecundity (**Figures 2A,C,D**) but in others the impact was negative (**Figure 2B**). As a non-pathogen, *Pfo* has been shown to induce PTI-related defense genes following infiltration into leaves of several plant species (Nguyen et al., 2010). We therefore made use of the *Pfo* EtHAn strain to assess ability of Me47 to suppress the PTI responses induced by this non-pathogenic bacterium. In these experiments, tomato plants were challenged with *Pfo* EtHAn delivering either GUS or Me47. The tomato defense genes *Lrr22* and *Pti5* have previously been shown to be inducible at 6 hpi following *Pfo* treatment (Nguyen et al., 2010). In our experiments, *Lrr22* and *Pti5* were only slightly induced by *Pfo* EtHAn (pVSP-GUS) but this increase was not statistically significant relative to the buffer control (**Figures 3A,B**). However, as the initial study used tomato cultivar Rio Grande-prf3, and the cultivar used in experiments described here is Moneymaker, it is possible that there is temporal variation in defense gene induction between tomato cultivars.

As we previously showed that Me47 can enhance aphid performance on tomato (**Figure 2D**), we suspected that Me47 might further suppress expression of these two defense genes. To our surprise, we found that delivery of Me47 by *Pfo* EtHAn enhanced the induction of both *Lrr22* and *Pti5* when transcript abundance was assessed at 6 hpi (**Figures 3A,B**, *p* < 0.05). For the PAMP-inducible gene *Gras2*, Nguyen et al. (2010) reported no induction at 6 hpi following *Pfo* infection. Similarly, we found no change in expression between the buffer and *Pfo* EtHAn (pVSP-GUS) treatments (**Figure 3C**). However, *Gras2* was significantly induced following *Pfo* EtHAn (pVSP-Me47) treatment (**Figure 3C**, *p* < 0.05). In this experiment, we also analyzed the expression of the *PR1a* reporter gene as it is frequently observed to be inducible both during pathogen infection and by PAMP treatment. Similar to the other genes tested, *PR1a* was highly induced following *Pfo* EtHAn (pVSP-Me47) treatment relative to both *Pfo* EtHAn (pVSP-GUS) and the buffer control (**Figure 3D**, *p* < 0.05). Together, this defense gene expression dataset illustrates the surprising observation that delivery of Me47 into tomato leaves enhances the expression of PAMP-responsive genes during bacterial challenge.

**FIGURE 4:**
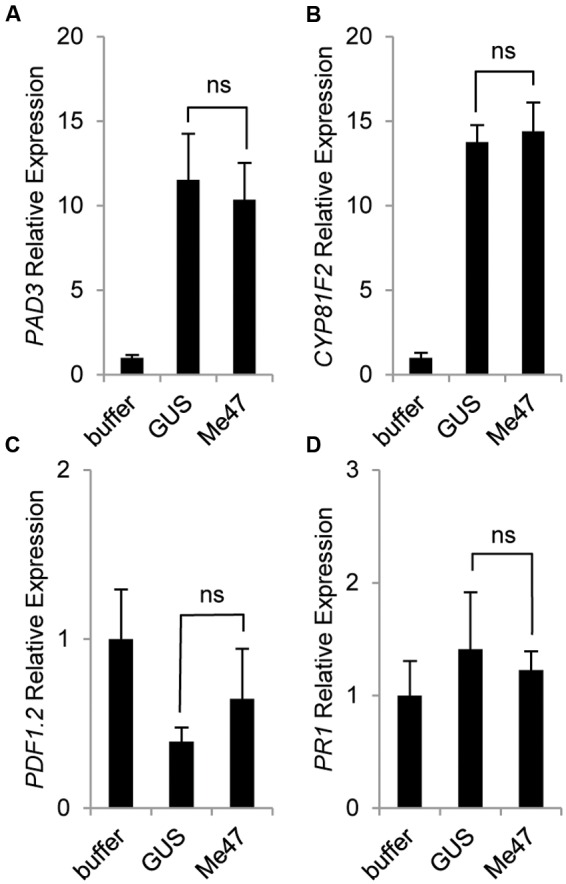
**Me47 has no effect on defense gene induction in *Arabidopsis*.** *Arabidopsis* Col-0 was treated as described in **Figure 3** and leaves harvested at 6 hpi. Expression analysis of genes involved in **(A)** camalexin (*PAD3*), **(B)** indole glucosinolate (*CYP81F2*), **(C)** Jasmonic acid/ethylene (JA/ET; *PDF1.2*) and **(D)** SA pathway (*PR1*) done by qRT-PCR. Bars represent means and standard error across six biological replicates from two independent experiments. Buffer treatment rescaled to 1 for presentation. Differences between GUS and Me47 treatments were not significant (ns).

To assess whether a similar phenomenon is present in another host used in our aphid bioassay, we conducted a parallel experiment to monitor defense gene induction in *Arabidopsis* following delivery of Me47 by *Pfo* EtHAn (**Figures 4A–D**). Unlike tomato, a specific defense marker assay for *Pfo* infection has not been developed for *Arabidopsis*. However, numerous studies have used *Arabidopsis* for dissection of aphid-relevant defense pathways (De Vos et al., 2005; Couldridge et al., 2007; Kusnierczyk et al., 2008; Kettles et al., 2013). The camalexin biosynthetic gene *PAD3* is known to be involved in resistance to numerous pathogens in addition to aphids. We therefore hypothesized it would be a good choice for assessing defense activation in plants challenged with *Pfo*. Whilst *Pfo* infection indeed caused a significant increase in *PAD3* expression (**Figure 4A**), there was no difference in expression levels between the *Pfo* EtHAn (pVSP-GUS) and *Pfo* EtHAn (pVSP-Me47) treatments at 6 hpi (**Figure 4A**). *CYP81F2* has been reported to be involved in the production of indolic glucosinolates that have activity against some pathogens and also aphids (Bednarek et al., 2009; Pfalz et al., 2009). Again, we found that expression of this gene was highly responsive to *Pfo* treatment at 6 hpi irrespective of the expressed construct (**Figure 4B**). *PDF1.2* is routinely used as a defense marker of specific relevance to the jasmonic acid (JA)/ethylene signaling pathways, whilst *PR1* has long been known to be highly responsive to many pathogens/pests and as a marker for salicylic acid (SA)-related defense signaling. Neither of these genes showed statistically significant responses either to *Pfo* treatment or delivery of Me47 relative to GUS at 6 hpi (**Figures 4C,D**). Together, we found no evidence of enhanced defense marker gene expression in *Arabidopsis* leaves treated with *Pfo* EtHAn (pVSP-Me47) relative to *Pfo* EtHAn (pVSP-GUS) at 6 hpi, thus illustrating differential responses of tomato and *Arabidopsis* to the Me47 effector protein.

### Me47 Is a Glutathione-*S*-Transferase with Activity against Isothiocyanates

**FIGURE 5:**
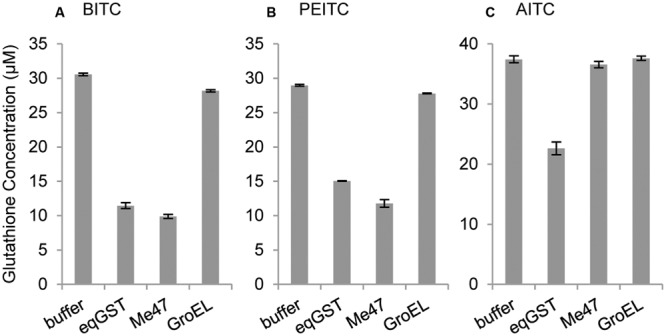
**Me47 can utilize selected isothiocyanates as substrates.** Glutathione depletion assay analysis of substrates **(A)** benzyl isothiocyanate (BITC), **(B)** phenylethyl isothiocyanate (PEITC), and **(C)** allyl isothiocyanate (AITC), incubated with buffer control, commercial equine GST (eqGST; Sigma), recombinant N-terminal His-tagged Me47 (Me47) and non-enzymatic recombinant N-terminal His-tagged GroEL (GroEL).

To confirm that Me47 is a functional GST, recombinant N-terminal His-tagged Me47 (His-Me47) was expressed and purified from bacterial cell lysates (Supplementary Figure S1) for use in a GST activity assay utilizing the broad-spectrum GST substrate 1-Chloro-2,4-dinitrobenzene (CDNB). Surprisingly, purified His-Me47 showed no ability to conjugate glutathione to CDNB when compared to commercially available GST preparations (data not shown). Nonetheless, we developed a glutathione depletion assay based on the method of Wadleigh and Yu (1988) to assess activity of Me47 against a selection of isothiocyanates (ITCs). These volatile defense compounds are specific to members of Brassicaceae, have toxic activity against insects and are known substrates for both insect and human GSTs (Wadleigh and Yu, 1988; Kolm et al., 1995). The bacterial chaperonin GroEL, also expressed and purified with an N-terminal His-tag (His-GroEL), was used as a negative control. In this assay, His-Me47 depleted the free glutathione in the presence of benzyl isothiocyanate (BITC; **Figure 5A**) and phenylethyl isothiocyanate (PEITC; **Figure 5B**) to a level comparable to the commercially prepared equine GST (eqGST) positive control. As expected, His-GroEL did not have any glutathione-depleting activity in the presence of either BITC or PEITC similar to the buffer-only control. In contrast, His-Me47 was unable to utilize allyl isothiocyanate (AITC) as substrate and the free glutathione level remained consistent with the buffer and His-GroEL protein controls (**Figure 5C**). These data illustrate that Me47 is a functional GST with ability to utilize known plant defense compounds as substrates.

### Me47 Does Not Interfere with the PAMP-Induced ROS Burst in *N. benthamiana*

The *M. persicae* effector Mp10 was previously demonstrated to suppress the flg22-induced ROS burst when transiently expressed in *N. benthamiana* (Bos et al., 2010). We conducted a similar experiment to test whether Me47 might have similar properties. In contrast to Mp10, Me47 had no suppressive effect on the flg22-induced ROS burst relative to leaf tissue expressing a GFP control transgene (Supplementary Figure S2A). Given that Me47 was found to induce PAMP-responsive defense genes when delivered into tomato leaves (**Figure 3**), we then tested whether Me47 might trigger a ROS burst in *N. benthamiana* using the same recombinant Me47 protein as used for the glutathione depletion assay (**Figure 5**, Supplementary Figure S2). Me47 protein was unable to induce ROS production above the PBS control levels (Supplementary Figure S2B) indicating that it is not an elicitor of ROS burst in *N. benthamiana*.

## Discussion

To date, only a handful of aphid effectors have been reported and for all the specific function or activity is unknown. For *M. euphorbiae*, effectors Me10 and Me23 are the only examples known to have impact on aphid fecundity when expressed *in planta* (Atamian et al., 2013). Agroexpression of both Me10 and Me23 increased *M. persicae* fecundity on *N. benthamiana*, whilst only Me10 increased potato aphid fecundity on tomato when delivered through bacterial T3SS (Atamian et al., 2013). In studies on *M. persicae* effectors, Mp10 and Mp42 were found to reduce aphid fecundity on *N. benthamiana* (Bos et al., 2010). Additional *M. persicae* effectors, Mp55-58, were described by Elzinga et al. (2014) that have either beneficial or deleterious impact on aphid success across *N. benthamiana, N. tabacum* or *Arabidopsis*. In each case, the change in aphid performance was consistent across the host species assayed. This indicates that well-conserved defense mechanisms may be subject to manipulation by these effectors.

Previously it has been also demonstrated that some aphid effectors have host-specific activity (Bos et al., 2010; Pitino and Hogenhout, 2013). For example, *M. persicae* effectors Mp1 and Mp2 enhanced *M. persicae* performance when stably expressed in *Arabidopsis*. In contrast, transient expression of the *A. pisum* orthologs of these genes had no effect on *M. persicae* performance. Me47 is the first aphid effector observed to have both beneficial and detrimental impact on aphid colonies that is host-dependent. In tomato, Me47 improved *M. euphorbiae* reproductive success (**Figures 2C,D**) yet Me47 protein or activity was recognized and induced the expression of multiple defense genes (**Figure 3**). Remarkably, Me47 decreased *M. persicae* fecundity on *Arabidopsis* (**Figure 2B**), yet there was no immune recognition of Me47 protein or activity, at an early effector delivery time point (6 hpi), as indicated by expression levels of several genes previously linked to aphid defense (**Figure 4**). These observations are surprising, as it is expected that an increase in aphid fecundity (as on tomato) would align with some degree of immune suppression and not immune activation. Furthermore, a decrease in aphid fecundity (as on *Arabidopsis*) might be expected to accompany a degree of immune stimulation. Together, these observations suggest that the direct recognition of either Me47 or its activity does not underpin the likelihood of successful aphid colonization. Additionally, the immunogenicity of Me47 may be suppressed in natural aphid infestations by the action of other, as yet unidentified, effector proteins present in the salivary milieu.

It has been speculated that aphids might actively trigger host defenses that have little efficacy against this class of plant attacker (Walling, 2008). For example, it is known that the JA and SA signaling pathways can act antagonistically where induction of one leads to suppression of the other. Aphid infestations primarily elicit SA-dependent defenses (De Vos et al., 2005; Kettles et al., 2013), yet other studies have reported JA-mediated defense to be more effective (Ellis et al., 2002; Zhu-Salzman et al., 2004). It is possible that the defense pathways triggered by Me47 delivery in tomato have little impact on aphid colonization, but might supress more effective defense responses not included as part of this investigation. Indeed, *PR1a* is frequently used as a marker gene for SA-dependent defense responses and was highly induced by Me47 in tomato (**Figure 3D**) but not in *Arabidopsis* (**Figure 4D**) consistent with this hypothesis.

Me47 delivery in tomato induces defense-related genes as determined by qRT-PCR (**Figure 3**). However, the elicitor activity of Me47 remains to be determined. From limited available data, it appears many endogenous *Arabidopsis* GSTs have non-specific subcellular localization and are present in the cytosol (Dixon et al., 2009), although a limited number are nuclear- or peroxisome-localized. We were unable to precisely localize Me47 in plant cells as transient expression of yellow fluorescent protein (YFP)-tagged Me47 in *N. benthamiana* revealed localization to both the cytosol and nucleus similar to YFP control (Data not shown). Since the Me47-YFP size is 57 kDa the protein could defuse through the nuclear pore. Nevertheless, it is unlikely that Me47 is present in plant organelles in the absence of endogenous GSTs. One possibility is that it is not Me47 that is recognized but the metabolomic products of its activity. Me47 substrate specificity may be different from endogenous GSTs, such that Me47-catalyzed reaction products are hallmarks of a foreign GST. Aberrant GST activity might therefore be open to recognition and stimulate immunity in a manner analogous to the perception of PAMPs during the PTI phase of host–microbe interactions.

A plethora of secondary plant metabolites exists and is speculated the primary function of many is for defense against herbivory. To overcome these defenses, insects have evolved large and diverse classes of detoxification enzymes, including GSTs, to negate the potentially lethal effects of toxic phytochemicals (Li et al., 2007; Ramsey et al., 2010). In aphids, GSTs have been linked to detoxification of glucosinolates (Francis et al., 2005) and nicotine (Ramsey et al., 2014) and the cereal hydroxamic acid 2,4-dihydroxy-7-methoxy-1,4-benzoxazin-3-one (DIMBOA; Mukanganyama et al., 2003). A single GST from the cotton bollworm (*Helicoverpa armigera*) was also found to detoxify the plant JA pathway precursor 12-oxophytodienoic acid (*cis*-OPDA; Dabrowska et al., 2009). However, the focus of insect GSTs has been almost exclusively on their role in the gut (for phytochemical detoxification) or in the cuticle/body (for insecticide detoxification; Vontas et al., 2001, 2002). To our knowledge, no prior study has described the role of a single GST, from any insect, out of the producing organism and in direct mediation of host–pest interactions. It is perhaps not surprising that such a mechanism has evolved in insects, as longer exposure time of toxic phytochemicals to detoxification enzymes likely reduces the concentration of toxin ingested and exposed to cells in the gut. Whilst this function for Me47 is therefore novel, it is unlikely to be the only example of such a phenomenon. Indeed, a catalytically active GST, expressed in salivary glands of wheat-infesting Hessian fly (*Mayetiola destructor*; Yoshiyama and Shukle, 2004) suggest that additional examples will exist in other groups of plant pests.

In our characterization of Me47, we found that Me47 substrate specificity did not include CDNB, a model substrate found to be metabolized by total GST preparations from *M. persicae* (Francis et al., 2005). The activity spectrum of total GST extracts from *M. euphorbiae* have not been described, but it is possible that other GSTs aside from Me47 show activity against this model substrate. In this study, we identified two ITCs (BITC, PEITC) as Me47 substrates (**Figure 5**). Me47 did not metabolize AITC, however, suggesting some degree of enzymatic specificity within this class of defensive metabolite. ITCs are defense compounds associated with insect resistance and are specific for cruciferous plants. During aphid infestations of plants belonging to this family, such as *Arabidopsis*, the function of Me47 is therefore clear. However, natural substrates of Me47 from the other hosts used in this study, tobacco and tomato, remain to be identified. It is therefore not yet possible to assess whether Me47 is a highly promiscuous, broad-spectrum GST or moderately promiscuous in its activity against plant defense compounds of relevance to natural *M. euphorbiae* infestations. Our initial data regarding metabolism of ITCs, coupled with the inability of Me47 to metabolize the model substrate CDNB would suggest the latter, but this requires further biochemical investigation.

## Author Contributions

GK and IK conceived and planned the experiments. GK performed the experiments. GK and IK analyzed the data. GK wrote the manuscript with help from IK.

## Conflict of Interest Statement

The authors declare that the research was conducted in the absence of any commercial or financial relationships that could be construed as a potential conflict of interest.
