# Supplementary figures and images for: The Potato Aphid Salivary Effector Me47 Is a Glutathione-S-Transferase Involved in Modifying Plant Responses to Aphid Infestation
Source: Front Plant Sci. 2016 Aug 3;7:1142. doi: 10.3389/fpls.2016.01142 (PMC4971587; doi:10.3389/fpls.2016.01142)

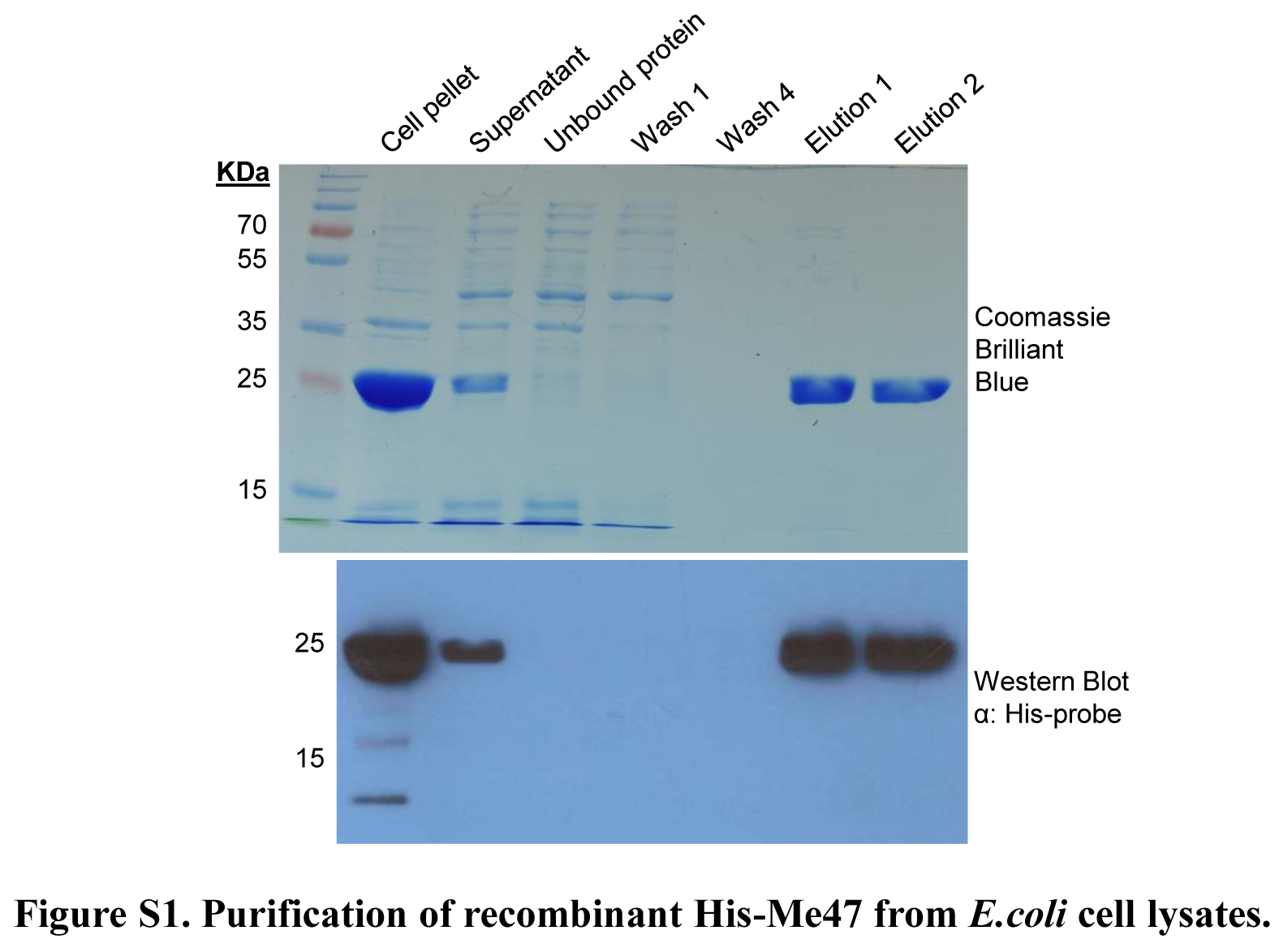

Supplement: Supplementary file 3 [file Image_1.TIF]

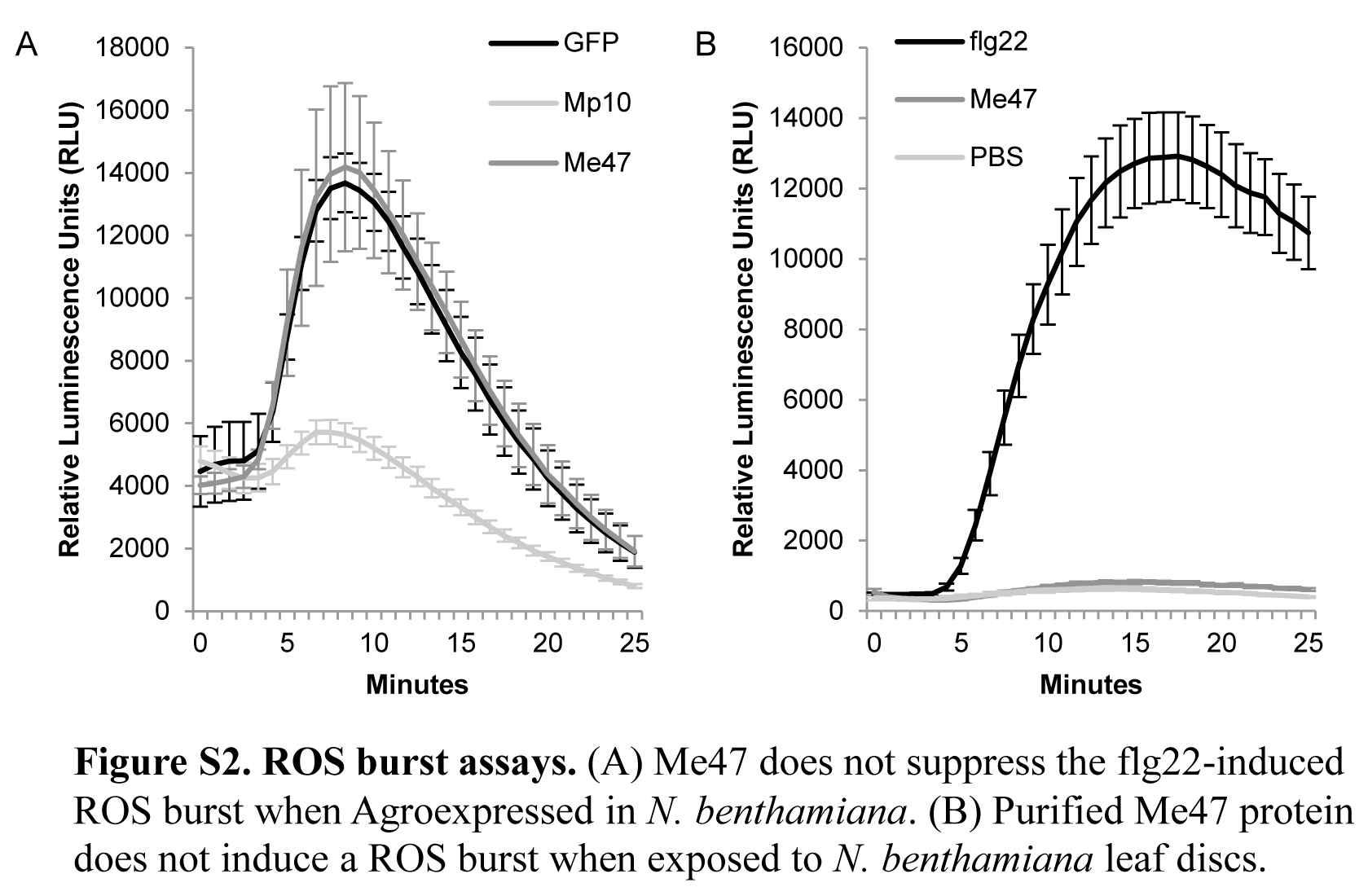

Supplement: Supplementary file 4 [file Image_2.TIF]

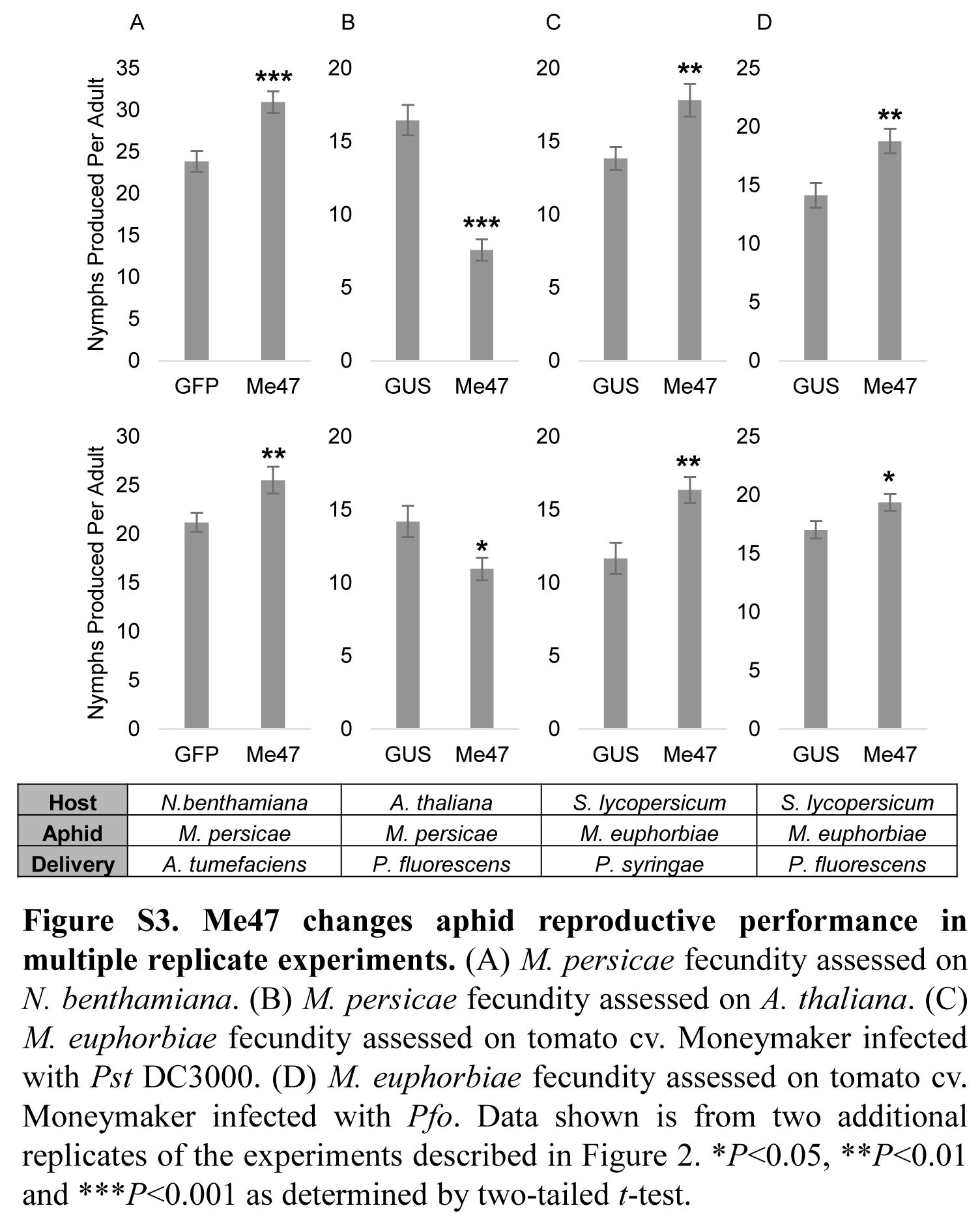

Supplement: Supplementary file 5 [file Image_3.TIF]
